# Warburg effect in colorectal cancer: the emerging roles in tumor microenvironment and therapeutic implications

**DOI:** 10.1186/s13045-022-01358-5

**Published:** 2022-11-01

**Authors:** Xinyang Zhong, Xuefeng He, Yaxian Wang, Zijuan Hu, Huixia Huang, Senlin Zhao, Ping Wei, Dawei Li

**Affiliations:** 1 Department of Colorectal Surgery, Fudan University Shanghai Cancer Center, Shanghai, 200032 China; 2 Department of Pathology, Fudan University Shanghai Cancer Center, Shanghai, 200032 China; 3 Cancer Institute, Fudan University Shanghai Cancer Center, Shanghai, China; 4 Institute of Pathology, Fudan University, Shanghai, China; 5 Department of Oncology, Shanghai Medical College Fudan University, Shanghai, China

**Keywords:** Colorectal cancer, Warburg effect, Metastasis, Tumor microenvironment, Therapeutics

## Abstract

Colorectal cancer (CRC) is the third most common cancer and the second leading cause of cancer-related death worldwide. Countless CRC patients undergo disease progression. As a hallmark of cancer, Warburg effect promotes cancer metastasis and remodels the tumor microenvironment, including promoting angiogenesis, immune suppression, cancer-associated fibroblasts formation and drug resistance. Targeting Warburg metabolism would be a promising method for the treatment of CRC. In this review, we summarize information about the roles of Warburg effect in tumor microenvironment to elucidate the mechanisms governing Warburg effect in CRC and to identify novel targets for therapy.

## Background

Cancer cells utilize lots of nutrients to sustain infinite proliferation and growth. This requires reprogramming of energy metabolism which is considered one of the hallmarks of cancer [1]. Moreover, alteration in energy metabolism leads to nutrition deficiency and metabolic waste accumulation, influencing the biological behavior of nearby non-tumor cells [2]. During the glycolysis process, cells break down glucose to produce pyruvate and a small amount of ATP. In normal cells with sufficient oxygen levels, pyruvate could enter the tricarboxylic acid (TCA) cycle to generate abundant energy whereas tumor cells exhibit high glycolysis activity regardless of the oxygen levels and produce lactate through activation of lactate dehydrogenase (LDH) and inhibition of pyruvate metabolism in mitochondria [3]. Such phenomenon was first observed by Otto H. Warburg in the early twentieth century and called the Warburg effect or aerobic glycolysis [4]. Aerobic glycolysis could meet the energy and nutrition demands essential for severe living conditions of tumor cells for cancer progression [3]. The role of glycolytic metabolism in cancer cells and nearby tumor microenvironment is complex and diverse. For example, enhanced glycolysis in cancerous cells relies on LDH-mediated production of NAD^+^ from NADH, reducing NADH:NAD^+^ ratio and suppressing p53 function [5]. In murine TNBC models, inhibition of glycolysis reduces the expression of cytokines such as granulocyte macrophage colony-stimulating factor (GM-CSF), granulocyte colony-stimulating factor (G-CSF) as well as the amount of myeloid-derived suppressor cells (MDSCs), further upregulating T cell immunity and inhibiting tumor development [6]. Herein, we summarize the oncogenic mechanisms of aerobic glycolysis, highlighting the latest developments and exploring the relation with some novel concepts.

Although various treatments can be used to treat colorectal cancer (CRC), the major concern that leads to CRC-related death nowadays is the metastasis of CRC [7]. Approximately half of the CRC patients could occur simultaneous or asynchronous metastases in liver, which becomes the most frequent metastatic organ in CRC [8, 9]. Surgical resection is suitable only for a small proportion of patients and chemotherapeutic treatment eventually leads to cancer progression due to initial or acquired resistance, highlighting the importance to develop new effective treatment [10–12]. The tumor microenvironment (TME) has rapidly gained attention in cancer research for the past several years. The tumor microenvironment includes the surrounding cellular environment around the tumor cells such as endothelial cells, immune cells, fibroblasts, mesenchymal stem cells (MSCs), and the extracellular matrix (ECM) [13]. A series of cytokines, chemokines, growth factors, exosomes, and other signaling molecules interact with each other and constitute a network within the TME to give tumor the ability to sustain and survive the increased stress, leading to cancer metastasis, immune suppression, abnormal angiogenesis, and drug resistance [13–15]. Abnormal glycolysis within TME can strongly impact the hallmarks of cancer and the function and composition of immune cells. For example, regulatory T (Treg) cells utilize lactic acid and promote the nuclear translocation of NFAT1, upregulating PD-1 expression in highly-glycolytic tumors [16]. Meanwhile, the impaired PD-1 expression in effector T cells leads to unsatisfactory results of immunotherapy [16]. Thus, it becomes important to explore the interplay between dysregulated metabolism and abnormal tumor immune microenvironment (TIME). In this passage, we summarized the influence of the Warburg effect on the metastatic ability of CRC and the role of Warburg effect in the microenvironment remodeling of colorectal cancer, mainly focusing our attention on glycolytic metabolism in immune cells. Further, we discuss the effect of glycolytic metabolism on CRC therapy to explore whether glycolysis-related enzymes, transporters, and transcription factors can be of therapeutic importance in cancer treatment. We summarize several relevant small-molecule inhibitors that have been used in preclinical and clinical trials to act as adjuvant therapy strategies, increasing the effectiveness of existing programs. Finally, we discuss the metabolic role of current therapeutic drugs in CRC, highlighting that glycolytic metabolism can be an important part of immunometabolism.

## Glycolytic metabolism and its regulation in cancer

### Glycolytic metabolism in cancer

**Fig. 1:**
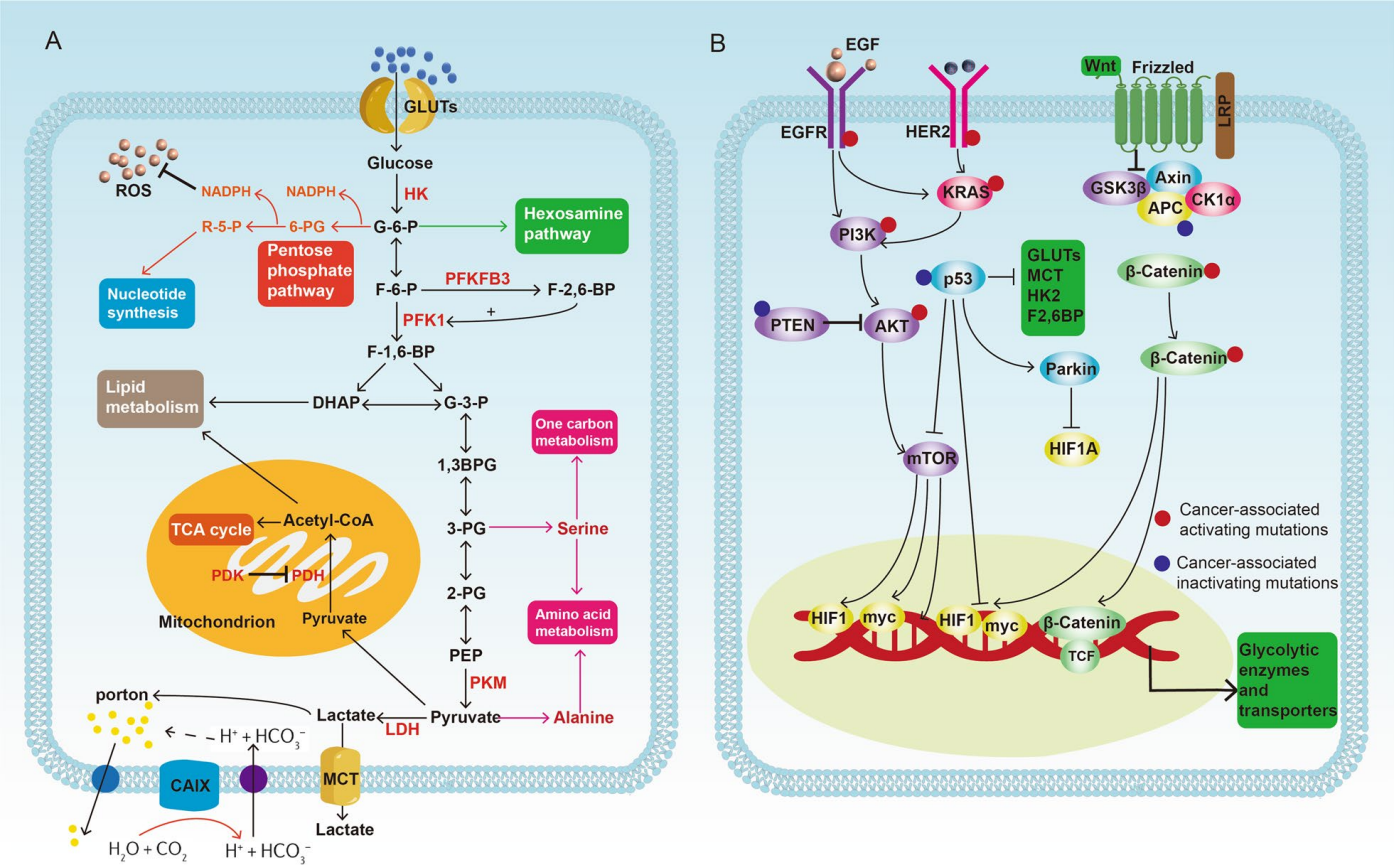
**A** Aerobic glycolysis in cancer. The transportation of extracellular glucose to the cytoplasm is achieved by glucose transporters (GLUTs). Hexokinase (HK) catalyzes the conversion of glucose to glucose-6-phosphate (G6P) and the conversion of Glucose-6-phosphate to fructose-6-phosphate (F6P) is a reversible reaction. The F6P can be catalyzed into fructose-1,6-biphosphate (F1,6BP) by phosphofructokinase1 (PFK-1). F1,6BP is converted into phosphoenolpyruvate (PEP) through a series of reversible reactions. Pyruvate kinase (PK) was responsible for catalyzing PEP into pyruvate and pyruvate can reversibly transformed to lactate by lactate dehydrogenase (LDH). Finally, lactate is transported out of cells which relies on the monocarboxylate transporter/MCT family. Carbonic anhydrases IX (CAIX) can export redundant protons and lactate and maintain the acid–base balance. In addition, the intermediates in glycolysis can enter other metabolic processes including hexosamine pathway, pentose phosphate pathway, lipid metabolism, TCA cycle, amino acid metabolism and one-carbon metabolism to synthesize biomacromolecules and meet the urgent growth needs of tumors. Arrows indicate positive modulations or transitions, while blunt ends indicate negative modulations. **B** The genetic phenotype in CRC regulates glycolysis. Mutations in APC lead to β-catenin/TCF transcriptional activation which induces increased transcription of β-catenin target genes to increase glycolysis. Inactivating mutations or deletion in the TP53 gene inhibits HIF1A, MYC, GLUTs, HK2, F2,6BP and MCT1 to reduce glycolysis. p53-induced upregulation of parkin can accelerate the degradation of HIF1. Activating mutations in RAS and the overexpression of EGFR trigger the activation of downstream pathways including PI3K/AKT/mTORC1 axis to promote glycolysis by enhancing glucose uptake, phosphorylating glycolytic enzymes and transcriptionally regulating glucose transporters and glycolytic enzymes expression via transcription factors such as HIF1A and MYC. Arrows indicate positive modulations or transitions, while blunt ends indicate negative modulations

The increased rate of glycolysis is a common metabolic change that occurs in cancer (Fig. 1A). It has been observed that active glycolysis in cancer is achieved by the upregulation of glycolytic enzymes and transporters. The initiation of the glycolysis process requires the transportation of extracellular glucose to the cytoplasm and this is achieved by sodium-glucose linked transporters (SGLTs) and facilitated diffusion glucose transporters (GLUTs) [17]. Next, a series of enzymes take part in aerobic glycolysis of which hexokinase (HK) catalyzes the conversion of glucose to glucose-6-phosphate (G6P), the first irreversible reaction in glycolysis [3]. Hexokinases (HKs) have five isoforms, namely HK1, HK2, HK3, HK4, and HKDC1 (hexokinase domain-containing protein 1) [18, 19]. Further, it has been reported that HK family genes were hypomethylated and exerted extensive CNV amplification in CRC [20]. Moreover, the expression of HK family genes was dysregulated and has been associated with survival in multiple kinds of cancers [20]. HK2 is the most well-characterized gene whose expression is significantly upregulated in many cancers such as prostate cancer, breast cancer, lung cancer, renal cancer, liver cancer and colorectal cancer [21–25]. Apart from performing metabolic functions, HK2 could also exert non-metabolic functions by binding to mitochondria to inhibit apoptosis and translocating into the nucleus to increase glucose uptake [26, 27].

Next is the conversion of glucose-6-phosphate to fructose-6-phosphate (F6P) which is a reversible reaction and the F6P can be catalyzed into fructose-1,6-biphosphate (F1,6BP) by phosphofructokinase1 (PFK-1), which is considered as the second rate-limiting step in glycolysis [28]. Multiple factors can regulate the activity of PFK1 among which fructose-2,6-bisphosphate (F2,6BP) is the most powerful allosteric activator [29]. The production of F2,6BP is completed by 6-phospho-fructo-2-kinase/fructose-2,6-bisphosphatase (PFKFB/PFK2), a bifunctional enzyme responsible for both degradation and synthesis of F2,6BP [30, 31]. PFKFB1, PFKFB2, PFKFB3, and PFKFB4 encode four different isozymes of PFK2 and the abnormal expressions of these isoenzymes are observed in a series of tumors except PFKFB1 [28, 32]. PFKFB3 has the lowest phosphatase/kinase ratio, thus has advantage to generate F2,6BP and increase the glycolytic flux of cancer cells [33]. Moreover, several oncogenic pathways, including Ras and mTOR pathways have been reported to regulate cancer metabolism by promoting the overexpression of PFKFB3 [34].

Further, F1,6BP is converted into 3-phosphoglycerate (3PG) via glyceraldehyde-3-phosphate (G3P), glycerate-1,3-diphosphate (G1,3DP) by aldolase and glyceraldehyde-3-phosphate dehydrogenase (GAPDH) [35]. Then, 3PG is converted to 2-phosphoglycerate (2PG) and phosphoenolpyruvate (PEP) through enolase [35]. Pyruvate kinase (PK) was responsible for catalyzing PEP into pyruvate, the final committed step of glycolysis. Pyruvate kinase (PK) has isoforms, namely liver PK (PKL), red blood cell PK (PKR), PKM1, and PKM2 [36]. The human PKM gene has 12 exons and could be alternatively spliced to produce different transcripts [37]. It has been studied that serine/arginine-rich splicing factor 3 (SRSF3) could remove exon 10 from PKM mRNA and generate PKM1 transcript while some oncogenic splicing factors such as heterogeneous nuclear ribonucleoprotein A1 and A2 (hnRNPA1, hnRNPA2) remove exon 9 to form PKM2 transcript [37]. However, the PKM1 exhibits a persistent high-glycolytic tetrameric form while PKM2 exists in either a low-glycolytic dimeric form or tetrameric form depending on the environment and cellular state [38]. PKM2 is overexpressed in the majority of cancers and promotes tumor development through various mechanisms, both metabolic and non-metabolic, and is the most deeply researched isoform of PK [36, 39].

The end product of glycolysis, pyruvate can enter the TCA cycle or be reversibly transformed to lactate by lactate dehydrogenase (LDH), which simultaneously oxidizes NADH to NAD^+^ [40]. In humans, the LDH family has a total of 5 isoforms and are tetrametric enzymes consisting of M and H subunits encoded by Ldh-A and Ldh-B, respectively [41, 42]. The more number of M subunits in tetramer will favor the glycolytic nature of LDH isoenzymes, indicating higher efficiency to convert pyruvate to lactate (LDH5/LDHA); conversely, LDH1/LDHB has four H subunits which favors the conversion of lactic acid to pyruvate, entering enter TCA cycle [41, 42]. However, the upregulation of LDHA can be found and is correlated with tumor progression in many cancers [41]. In addition, the upregulated LDHA level is considered a prognostic factor in a series of cancers, such as pancreatic cancer, breast cancer, renal cancer, lung cancer, and liver cancer [40, 43–48]. Finally, lactate is transported out of cells which relies on the monocarboxylate transporter/MCT family [49]. Meanwhile, varying degrees of MCT family upregulation were observed in tumors to adjust massive production of lactic acid, thus avoiding or mitigating tumor acidosis [49].

Elevated glycolytic metabolism is a common event in cancer cells because tumors undergo competition due to limited and shared nutrients with stromal cells and the immune compartment and a higher rate but lower yield of ATP production achieved by glycolysis could gain a selective advantage [50–52]. Thus, cancer cells can fasten their glycolysis rate and compensate for the energy gap. This phenomenon is so conspicuous that positron emission tomography (PET) can diagnose the recurrence and metastasis of cancer [53, 54]. High consumption of glucose by cancer cells significantly reduces its availability in TME, forming a low-glucose extracellular environment and disturbing the function of immune cells [55]. Meanwhile, cancer cells secret abundant glycolysis-derived lactate, resulting in acidosis in microenvironment. Lactate is not a matter of metabolic waste. Actually, lactate can be further utilized by some non-tumor cells and a proportion of tumor cells [56]. Acidic TME also promotes local invasion, metastasis and dampens the anti-tumor function of immune cells [56–58]. Further, lactate can take part in signal transduction by functioning as an intracellular mediator or an extracellular ligand to bind to some receptor such as GPR81 [56, 59].

The Warburg effect is also regarded as an adaptative mechanism besides producing lactic acid to synthesize biomacromolecules and meet the urgent growth needs of tumors. The glucose in the tumor microenvironment can be used as a carbon source for anabolic processes such as the de novo synthesis of nucleotides, lipids, and proteins [60–62]. For instance, G6P can further enter into pentose phosphate pathway (PPP) to promote the synthesis of ribose, an indispensable ingredient of nucleotide. At the same time, this process allows cancer cells to transform NADP + into NADPH, which is an essential coenzyme in lipid metabolism [60]. 3PG and pyruvate can be transformed into serine and alanine, respectively. Serine can further take part in one-carbon metabolism and produce glutathione and NAPDH [55]. NAPDH also plays an important role in maintaining redox homeostasis by upregulating the level of GSH. Hence, Warburg effect can enhance the synthesis of NADPH to encounter the excessive oxidative stress in tumor cells [55, 63]. Apart from NADP + and NADPH, NADH and NAD + are also important factors that are used to transport electron in the mitochondria to regulate redox potential in tumor cells. Conversion from pyruvate to lactate requires NADH, which regenerates NAD + to avoid the excessive accumulation of NADH [2].

Glycolytic metabolism also influences epigenetic modifications in cancer [64]. Pyruvate that derived from glucose could be transformed into acetyl-CoA, which could acetylate histones and further regulate the transcription of certain DNA [64, 65]. Histone acetylation makes it easier for transcription factors to bind to DNA and promote cell growth [66, 67]. Histone acetylation is easily affected by cellular signaling and the nutrition status of cells [66]. Cells with high glycolysis rate often maintain the NAD + /NADH ratio at a low level, which could enhance histone acetylation by inhibiting the activity of sirtuins [68, 69]. When nutrient is deprived, NAD + level can be upregulated which functions as a cofactor of sirtuins to promote deacetylation [70]. As for methylation, high level of 3PG increases the synthesis of serine. Serine could regulate methylation by linking with the folate cycle, which is coupled to the methionine cycle [64]. Recently, a novel epigenetic regulation termed histone lactylation has been reported by Zhang et al. and the study revealed that lactate-derived lactylation of histone lysine (K) residue can promote gene transcription from chromatin [71]. These findings suggest that glycolytic metabolism significantly affects the epigenetic landscape of tumor.

### Regulation of aerobic glycolysis

#### Transcriptional regulation of glycolysis

Increased activities of glycolysis rely on the upregulation of enzymes in the pathway which are mainly regulated by hypoxia-inducible factor 1 (HIF1) and c-myc. HIF-1 is a heterodimeric transcription factor and is composed of a HIF-1α subunit that senses the changed oxygen levels and a HIF-1β subunit that is constitutively expressed [72]. Under normoxia, tumor suppressor von Hippel–Lindau protein (VHL) ubiquitylates HIF-1α, enabling the activation of ubiquitin ligase system and subsequent proteasomal degradation of HIF1α [72, 73]. In the absence of oxygen, HIF-1α is stabilized and binds to HIF-1β to form a heterodimer which further enters into the nucleus and transcriptionally activates HIF-1–targeted genes [72, 73]. Besides the heterogeneous oxygen concentration inside the tumor, oncogene activation (e.g., RAS-induced mTOR) or tumor suppressor inactivation (e.g., p53, PTEN) can enhance HIF1α expression, suggesting modulation of HIF1α through diverging mechanisms [74]. HIF1α promotes the expression of glycolytic enzymes including HK2, aldolase, LDHA, and glycolytic transporters such as MCT4, and GLUT1 and increases the intracellular level of glycolysis [75, 76].

MYC is recognized as one of the most frequently amplified oncogenes in human cancers [77]. The product, myc, can interact with Max and form a heterodimer that binds to E box-containing gene promoters and is widely involved in the regulation of genes [78, 79]. Moreover, myc could upregulate the majority of the key glycolytic enzymes and transport proteins such as GLUT, LDH, and MCT1 to activate aerobic glycolysis [77, 80]. Furthermore, oncogenic splicing factors such as hnRNPA1 and hnRNPA2 can also be induced by MYC to sustain a high level of PKM2/PKM1 ratio in cancer cells [81].

#### The genetic phenotype in CRC regulates glycolysis

Most colorectal cancer evolves in a decade time from an aberrant crypt to a polyp (neoplastic precursor lesion), and finally becoming colorectal cancer [7]. The adenoma-carcinoma-metastasis model relies on the accumulation of genetic events of “APC-KRAS-TP53,” which is also known as the Vogelstein model [82]. This classical theory highlights the importance of these genes (APC, KRAS, TP53) in the development of the majority of CRC, which can activate oncogenic signaling pathways and the downstream transcriptional factors, ensuring highly proliferative cancerous cells [83]. Herein, we focus on the role of genetic mutations in regulating the glycolytic metabolism of CRC, highlighting the importance of the gene-signaling pathway-transcriptional factor axis in glycolysis (Fig. 1B).

A metabolic signature toward glycolysis was found in the early stage of CRC [84]. Meanwhile, the APC gene was found to be frequently (> 80%) mutated in sporadic colorectal cancers, which partly promotes tumorigenesis by enhancing glycolysis in CRC [85, 86]. Moreover, the transcriptome analysis has indicated that the murine CRC model with mutated APC gene has a more glycolytic phenotype which supports tumorigenesis [87]. Further, alterations in APC lead to β-catenin/TCF transcriptional activation which induces increased transcription of β-catenin target genes, including cMYC, PKM2, and pyruvate dehydrogenase kinase 1 (PDK1) and monocarboxylate transporter 1 (MCT1) [85, 86, 88, 89]. PDK1 is a glycolysis-promoting enzyme that could reduce the conversion of pyruvate into acetyl-CoA and inhibit the mitochondrial oxidative phosphorylation (OXPHOS), thereby maintaining aerobic glycolysis in tumor cells [90]. Two transcription factors, HIF1α and MYC, are also regulated by APC/β-catenin axis [89]. In colorectal cancer, APC mutation leads to the accumulation of β-catenin/Tcf4 complex, which could bind to the promoter of c-MYC to indirectly induce glycolysis [89]. Also, aberrant activation of β-catenin could enhance HIF-1α-induced glucose metabolic reprogramming in CRC [91–93].

Inactivating mutations or deletion in the TP53 gene happens in 40%-50% of sporadic CRC and occurs in 80% of the advanced CRC [94, 95]. As a well-known tumor-suppressor gene, p53 negatively regulates the metabolism of glycolysis and restrains the tumor cells from metabolic plasticity [96]. Mechanically, p53 suppresses the translocation of GLUT1 to the plasma membrane, transcriptionally inhibits GLUT1, GLUT3, and GLUT4, and reduces the glucose uptake [97–99]. Moreover, p53 could directly or indirectly regulate the expression of glycolytic enzymes such as HK2 and F2,6BP and downregulate the rate of glycolysis [100]. Additionally, p53 can downregulate the expression of MCT1 to reduce intercellular lactate transportation [101]. P53 also has close interactions with HIF-1α and MYC [102]. HIF-1 can suppress p53 expression in normal cells with mild hypoxia. In reverse, HIF-1 transactivation can also be attenuated by p53 because p53 can compete for p300 transcriptional cofactor, which is essential to the transcriptional activity of HIF-1α [102, 103]. p53 can also induce the expression of parkin, a transcriptional product of PARK2 [104]. p53-induced upregulation of parkin has been reported to accelerate the degradation of HIF1α by directly binding to its ubiquitination site [105]. P53 also transcriptionally represses c-myc by decreasing histone acetylation level at the promoter of c-myc and recruiting corepressor to the c-myc promoter [106]. Hence, in p53 mutant cancer cells, the inhibitory effect of p53 on HIF1α and MYC was eliminated, leading to HIF1α and MYC accumulation.

Oncogenic mutations occur and accumulate in tumors and making their independence and less influenced by extracellular stimuli compared with normal cells [107, 108]. About half of the CRC patients have activating mutations in RAS which lead to continuous activation of phosphoinositide 3 kinase (PI3K)/Akt/mechanistic target of rapamycin complex 1 (mTORC1) pathway [83]. Despite the low EGFR mutation rate (1%) that exists in CRC, the overexpression of EGFR is found in 80% of CRC cases which triggers the activation of downstream pathways including PI3K/AKT [109]. RAS mutation and EGFR overexpression together consistently activate PI3K/AKT which is a major event leading to aberrant cancer metabolism [110]. AKT and mTORC1 are two pivotal regulators of metabolic signaling events integrating metabolite availability and growth factor signaling. The role of AKT and mTORC1 in metabolism has been extensively described in some reviews [110, 111]. In brief, AKT and mTORC1 promote glycolysis by enhancing glucose uptake, phosphorylating glycolytic enzymes. Further, PI3K–AKT pathway can transcriptionally upregulate the mRNA level of MYC and enhance the protein level of both HIF1α and MYC by inhibiting their degradation and promoting their translation [110, 111].

#### Epigenetic regulation of the Warburg effect

Epigenetic modifications, especially methylation, have been reported to regulate glycolytic metabolism [64]. Methylation regulates glycolytic metabolism by changing the activity and status of DNA, RNA and proteins. DNA methylation is responsible for the aberrantly activated glycolytic metabolism in cancer [112]. For instance, the promoter of LDHB is hypermethylation in cancer cells, which could upregulate the LDHA/LDHB ratio and further promote the production of lactate [112]. Also, the upregulation of HK2 can be mediated by the hypomethylation of its promoter, which promotes HK2 expression and tumor progression [113]. Further, the expression of HIF-1α and the activity of HIF pathway can also be upregulated by DNA methylation in cancer [112]. At the RNA level, N6-methyladenosine (m6A) modifications of RNAs take part in RNA metabolism to regulate the level of mRNA [114]. In CRC, METTL3 mediates the m6A modification of HK2 and SLC2A1 mRNA, which leads to their stabilization and activates the glycolytic pathway [115]. Furthermore, glycolytic enzymes such as LDHA and PKM2 can be methylated by methyltransferase, which can also change their activity [116, 117]. Hence, methylation plays an important role in regulating the glycolytic metabolism of cancer cells.

## The role of glycolysis in colorectal cancer liver metastasis (CRLM)

**Fig. 2:**
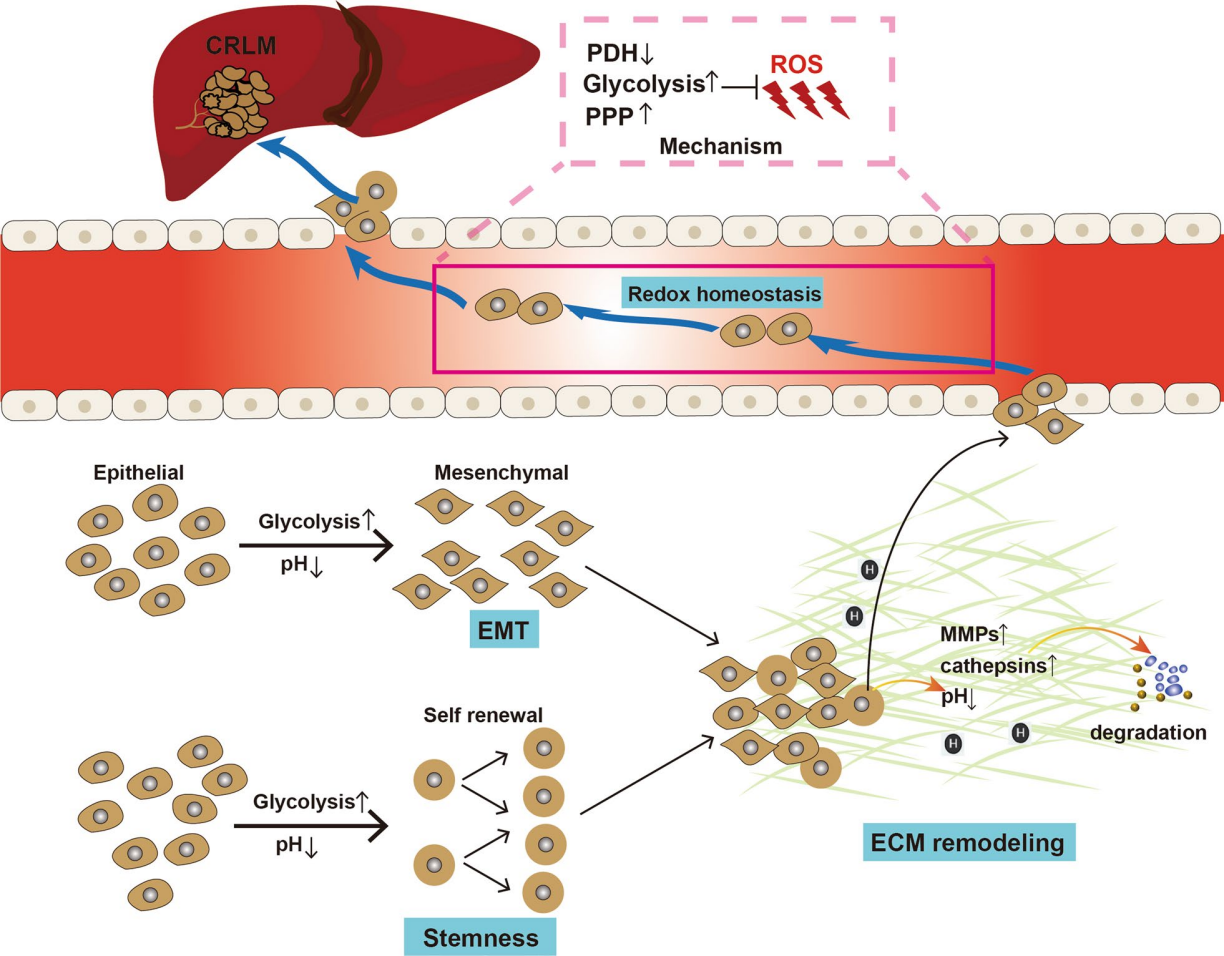
How glycolytic metabolism supports CRLM. Colorectal cancer liver metastasis (CRLM) relies on a small proportion of CRC cells which acquire a series of features including epithelial-to-mesenchymal transition (EMT), cell migration through extracellular matrix (ECM), stemness and redox homeostasis. Aerobic glycolysis could support these processes to accelerate metastasis. Arrows indicate positive modulations or transitions, while blunt ends indicate negative modulations

CRLM relies on a small proportion of CRC cells which acquire a series of features such as epithelial-to-mesenchymal transition (EMT), cell migration through ECM, survival in the blood to escape from the primary site and successfully settle in the liver to finally proliferate and termed as invasion-metastasis cascade (Fig. 2). Cancer cells dynamically change their metabolic features to adjust to different extracellular microenvironment, to meet their energy needs and to support metabolic plasticity [58]. Throughout invasion-metastasis cascade, cancer cells exhibit metabolic plasticity, which means that they can rely on the metabolic phenotype to support different metabolic requirements [58, 118]. Glycolytic metabolism is widely involved in metabolic plasticity to help tumor cells fuel their invasion and migration ability [58]. On the one hand, glycolytic metabolism upregulates the production of some metabolites, such as pyruvate and lactate, to synthesis necessary nutrients or change the extracellular environment. On the other hand, the glycolysis-related enzymes, transporters and transcription factors exert their metabolic and non-metabolic functions to induce various signaling pathways and malignant phenotypes.

Emerging evidence shows that glycolytic metabolism has a close connection to EMT which promotes a more aggressive phenotype in cancer cells and ultimately cause distant metastasis. Using a large-scale gene expression matrix, Justin Guinney et al. divided CRC into four different subtypes and the CMS4 subtype (23%) was considered as a mesenchymal phenotype which occupies a higher proportion of CRC with more advanced stages [119]. Increasing evidence has suggested that enhanced flux of glycolysis could induce a “mesenchymal phenotype” in CRC cells. Cancer cells treated with lactate have been reported to express enhanced mesenchymal epithelial markers and reduced epithelial markers in multiple kinds of cancers. In colorectal cancer, aerobic glycolysis induces high lactate concentration and is reported to promote the mesenchymal characteristics of the CRC cell line, indicating that acidic environment is favorable to cancer metastasis [120]. Further, enhanced glycolysis is accompanied by the upregulation of relevant enzymes and transporters. Ham et al. explored that nuclear PKM2 could suppress the E-cadherin expression by binding with TGF-β-induced factor homeobox 2 (TGIF2) and HDAC3 to induce the deacetylation of histone H3 in the promotor sequence of E-cadherin in colon cancer cells [121]. Overexpression of Glut3, HK3, GAPDH, aldolase A and B were also reported to promote the EMT of cancer cells through diverse mechanisms in CRC [122–127]. HIF1α, the key element of glycolytic metabolism, directly promotes EMT progression by controlling the expression of ZEB which is an EMT regulator [128]. Mechanically, the upstream promoter of ZEB1 contains hypoxia response element (HRE) sites where HIF1α binds and enhances the relevant gene transcription [128]. In addition, HIF1α overexpression has indirect mechanisms to induce EMT. For example, HIF-1α could bind to the HRE site in the promoter region of SRGN and enhance the EMT-activating ability [129]. In colorectal cancer, the most commonly activated signaling pathways, including the Wnt/β-catenin pathway and TGF-β pathway, play an important role in EMT formation [130]. HIF1α could also indirectly induce EMT by activating oncogenic signaling pathways such as the WNT/β-catenin pathway [131].

Several other factors like extracellular matrix degradation, reorganization of the cytoskeleton, and cell adhesion to the extracellular matrix are necessary for tumor cell migration [132]. Multiple studies have demonstrated that acidic pH upregulates the rate of migration in tumor cells. Warburg effect endows cancer cells with the ability to produce lactate under normoxia conditions. Mechanically, intracellular lactate can be exported to the outside of the tumor cells by MCTs transporters, resulting in the acidification of the microenvironment [133]. In addition, upregulation of HIF1A promotes the transcription of carbonic anhydrase IX and XII (CAIX, CAXII), which catalyze hydrated CO_2_ into bicarbonate and H^+^ ions. Accumulated intracellular H^+^ ions can be exported outside to further increase the extracellular acidity [134, 135]. The invasion of cancer cells into surrounding tissue depends on the digestion of extracellular matrix which is executed by a series of ECM-degrading proteases such as matrix metalloproteinases (MMPs) and cathepsins [136, 137]. CRC patients with distant metastasis have significantly higher levels of MMPs than non-metastatic CRC patients [138]. Several findings have indicated that the expression and activity of protases can be regulated by extracellular acidity [139]. Moreover, in colon cancer an increased activity of LDHA induces overproduction of lactate and enhances the secretion of metalloproteinases, which can be reversed by LDH inhibitors [140]. Meanwhile, cytoskeletal reorganization leads to a repeated and coordinated cycle of protrusion of the lamellipodium and retraction of the back of the cell that requires cell–matrix interactions mediated by integrins [141, 142]. Li et al. found that acidic pH could trigger cell protrusion and cytoskeletal dynamics via integrin β1-activation of the FAK-Src signaling [143]. Additionally, a moderately decreased pH can remodel cell-substrate adhesions and it has been reported that human cells can migrate faster at a pH of 7.0 compared to a pH of 7.4, suggesting lower pH could upregulate the dynamics of integrin-ECM attachments [144, 145].

Attachment of cells to ECM provides pro-growth and pro-survival signals to epithelial cells [146]. Tumor metastasis means a detachment of tumor cells from the ECM and subsequent entry into blood stream, which leads to the induction of cell death through several mechanisms, including anoikis and harsh oxidative stress [146]. A proportion of pyruvate could enter into TCA cycle and be catalyzed into acetyl-CoA by pyruvate dehydrogenase (PDH), a gatekeeper that controls the pyruvate flux into mitochondria [147]. In non-transformed cells, ECM detachment caused a decreased rate of PDH flux and TCA cycle while in ECM-detached cancer cells, the decrease in PDH flux can be restored by oncogenes, thus helping cancerous cells generate sufficient energy [148]. However, OXPHOS can enhance oxidative stress and lead to elevated reactive oxygen species (ROS) levels, irreversibly damaging cellular macromolecular components and causing cell death [148]. Metastasizing cancer cells can inhibit the expression of PDH through activating PDKs, which can downregulate TCA flux [147]. A restrained TCA flux reduces the overproduction of ROS and makes more pyruvate enter into glycolytic pathway. Pyruvate and lactate could contribute to the resistance to ROS in cancer cells [147, 149]. In patients with metastatic CRC, a higher serum lactate level can be found than non-metastatic CRC patients [58]. Intracellular pyruvate or lactate can enhance the expression of HIF1α to remodel a hypoxia environment, which is helpful to reduce ROS [63, 150]. Furthermore, upregulated pyruvate can enhance the branching pentose phosphate pathway and the production of NADPH, which is important for the antioxidant activity of tumor cells [151, 152]. Several studies have reported that a series of developed drugs inhibit the proliferation and metastasis of CRC via enhancing ROS levels, suggesting that targeting ROS has promising potential in cancer treatment [153, 154]. Overall, Warburg metabolism can uncouple oxidative and fermentative glucose metabolism to generate ATP at a faster rate and avoid excessive ROS generation [155, 156].

Cancer stem cells (CSCs) are a small subpopulation of malignant tumor cells characterized by tumorigenic properties and the ability to self-renew and form differentiated progeny, which can be characterized by several markers such as aldehyde dehydrogenase (ALDH), CD44 and CD133 [157, 158]. CSCs can exhibit high levels of OXPHOS or glycolysis, which is dependent on cancer type and extracellular environment [159]. In several types of cancers such as CRC, osteosarcoma, lung cancer and breast cancer, studies have indicated that CSCs exhibit higher glycolytic activity compared to their non-stemness counterparts [159]. Highly glycolytic CSCs enhanced their ability to uptake glucose and export lactate, accompanied by the upregulation of glycolysis-related proteins such as HK2, PKM2 and LDHA [159, 160]. The glycolytic metabolism in CSCs is regulated by multiple pathways. MYC plays an important role in maintaining the stemness features of CSCs, which could also enhance the expression of glycolytic enzymes [159]. HIF signaling can maintain the activation of Wnt/β-catenin signaling pathways and the stemness of colorectal cancer [161]. In addition, stemness markers CD44 and ALDH have been reported to promote glycolytic metabolism, further demonstrating the close interaction between stemness and glycolysis [162, 163]. Several studies have revealed the importance of aerobic glycolysis in maintaining the stemness and proliferation of colorectal cancer stem cells. In colon cancer, the secretomes of CSCs and isogenic differentiated tumor cells were analyzed. Compared to its differentiated counterpart, CSC-enriched proteins contain a series of glycolysis-related enzymes such as GPI, PGM1, and PGM2, indicating a preference for aerobic glycolysis to maintain their oncogenic function [164]. In addition, the increased glucose concentration increases the percentage of colon cancer stem cells in a time-dependent manner [165]. Further, it has been observed that 3-BrOP treated cells which is a glycolysis inhibitor could significantly reduce the percentage of stem cells and inhibit tumor development [165]. Some oncogenic mutations could control the glycolytic metabolism of CSCs in order to drive cancer initiation and progression and could be a potential target for cancer therapy. By increasing LDHA activity and subsequently aerobic glycolysis, an overexpressed adenylate kinase hCINAP enzyme in CRC can enhance invasion, metastasis, and self-renewal in colorectal cancer stem cells. In contrast, the depletion of hCINAP leads to inhibition of invasion, metastasis, self-renewal, and EMT in colorectal CSCs [166]. Considering a greater need for glycolysis exists in CSCs, therefore, a proper inhibition of this metabolic requirement might be a powerful weapon to damage CSCs and overcome the most intractable problem of drug resistance in cancer therapy.

## Crosstalk between the TME and glycolytic metabolism

**Fig. 3:**
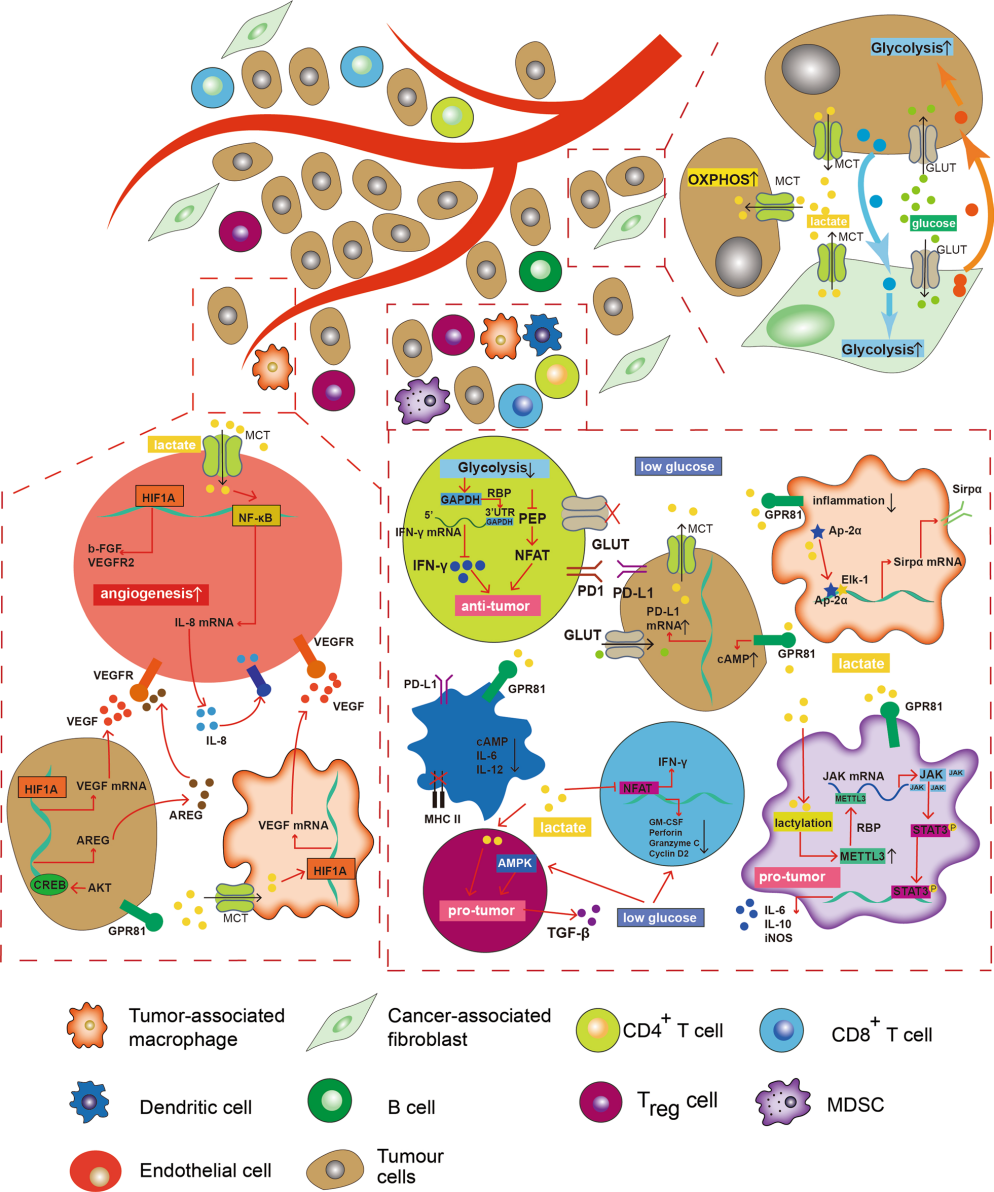
Glycolytic metabolism remodels tumor microenvironment. Lactate can promote tumor cells and tumor-associated macrophages (TAMs) to secret a series of factors to support angiogenesis. Endothelial cells can also sense the extracellular lactate level to promote their proliferation. Glucose deprivation and extracellular acidosis significantly suppresses the anti-tumor function of macrophages, CD4^+^ T cells, CD8^+^ T cells and dendritic cells (DCs) while has little influence on immunosuppressive cells such as myeloid-derived suppressor cells (MDSCs) and regulatory T cells (Tregs). Carcinoma-associated fibroblasts (CAFs) and cancer cells can promote the glycolytic levels of each other. Additionally, a part of tumor cells can uptake lactate and display oxidative metabolism, also known as “the reverse Warburg effect.” Arrows indicate positive modulations or transitions, while blunt ends indicate negative modulations

The cancer cell is not isolated. It communicates with surrounding stromal cells, immune cells, and other cancer cells all the time and senses changes in the extracellular environment, thus making corresponding adjustments (Fig. 3). Interactions among these cells increase tumor metabolism diversity and make cancer cells “guide” non-tumor cells and form a coexistence ecosystem [2, 167]. On the other hand, a heterogeneous tumor microenvironment leads to hypoxia, extracellular acidosis, and nutrition deprivation, significantly changing the proportion of immune cells and forcing stromal and immune cells to perform metabolic reprogramming [2, 168–170]. Hence, exploring the tumor microenvironment can offer therapeutic benefits.

### Warburg effect induces pathological angiogenesis

The tumor endothelial cells (TECs) are located next to the bloodstream but predominantly produce ATP via aerobic glycolysis to meet their emergent growth needs [171, 172]. Contrast to tumor cells whose enhanced glycolysis relies largely on the oncogenic alternations, endothelial cells are more susceptible to extracellular signals and metabolites, which means their glycolytic phenotype and proliferative ability can be controlled by tumor cells [173]. For example, an elevated level of HIF1α in cancer cells could subsequently activate the transcription of vascular endothelial growth factor (VEGF) which promotes the formation of new blood vessels. Enhanced aerobic glycolysis induces excessive production of lactate by both cancer cells and endothelial cells, leading to the extracellular accumulation of lactate [172, 173]. Moreover, elevated lactate production could be transported into endothelial cells to promote the formation of new blood vessels [171, 174]. Under normoxia, lactate was transported into endothelial cells through the MCT1 transporter and induced the activation of HIF-1 which could enhance the expression of basic fibroblast growth factor (bFGF) and vascular endothelial growth factor receptor 2/VEGFR2 to induce angiogenesis [175]. Intracellular lactate can also drive the phosphorylation or degradation of IκBα, thus stimulating the expression of NF-κB [176]. NF-κB activation could further induce the upregulation of IL-8 to support angiogenesis and tumor growth in CRC cells line [176]. Meanwhile, extracellular lactate can function as a signal molecule that could activate the G-protein coupled receptor GPR81 and it has been observed that the expression of GPR81 is upregulated in multiple kinds of cancers including colon cancer [177]. Lactate-induced GPR81 activates the downstream PI3K/Akt-CREB pathway, enhancing AREG production which is a pivotal protein in GPCR-induced angiogenesis [178]. Further, lactate could stimulate macrophages to secret a series pro-angiogenesis factors and regulate the growth of endothelial cells indirectly [179]. Recent studies have indicated that tumor-associated macrophages (TAMs) can take up lactate through their MCTs, followed by the lactate-induced activation of HIF-α, thus enhancing transcriptions of VEGF [180, 181]. In colon cancer, the inhibition of LDHA significantly reduced the extracellular level of lactate which leads to an inhibition of TAM-derived VEGF and tube formation [181].

### Warburg effect in the immune-suppressive tumor microenvironment

Immune cells undergo metabolic change to support their anti-tumor effect. Like tumor cells, some proliferative immune cells apply aerobic glycolysis as one of their metabolism programs to promote their rapid growth [168]. Antitumor CD4^+^ T cells and CD8^+^ cells are indispensable for the human adaptive system and their anti-tumor function relies on aerobic glycolysis [182]. Early upregulation of aerobic glycolysis in T cells is mediated by TCR signaling which promotes PDK1 activity in a transcription-independent manner [183]. PDK1 could phosphorylate and inhibit PDH, a gatekeeper that controls the pyruvate flux into mitochondria and promote the metabolic shift from the TCA cycle to glycolysis [183]. CD4^+^ T and CD8^+^ T cells further maintain their upregulated glycolysis level through CD28-mediated activation of PI3K–AKT signaling [183–185]. Activated CD8 + T cells upregulate the cell membrane expression of glucose transporters, especially GLUT1, to increase the transportation of glucose into cells to meet the growth needs [183, 185]. Besides CD28 co-stimulation, insulin, adipokine leptin and some cytokines such as IL-2 and IL-7 can also induce GLUT1 expression through activating AKT in CD8 + T cells [186]. It has been noted that the transcriptional regulation of aerobic glycolysis is mediated by MYC and HIF on activation of anti-tumor T cells [187, 188]. MYC and HIF work independently and facilitate the transcription of glycolysis-related enzymes and transporters. Glycolytic metabolism also plays a central role in supporting the anti-tumor function of effector T cells. Upregulated glycolytic level in both CD4^+^ T and CD8^+^ T cells can activate branching pathways such as PPP and serine-one carbon pathway to generate enough intermediates to support biosynthesis and their anti-tumor functions. For instance, enhanced flux of PPP promotes the generation of NADPH, which is essential to lipid metabolism and membrane synthesis in CD8 + T cells [189]. In activated CD8 + T cells, glycolysis-derived acetyl-CoA can enter into mevalonate biosynthetic pathway, providing essential components for the synthesis of sterols and ubiquinone, and substrates for protein isoprenylation [190]. Glycolytic enzymes can also function as RNA-binding proteins to bind to the mRNA of several cytokines, which was first reported in CD4 T cells where GAPDH binds to the mRNA of cytokines such as TNFA, IFNG and IL-2 and controls their translocation [191]. In quiescent CD8 + T cells, LDH can bind to the 3’UTR of TNF, IFN-γ and IL-2 mRNA [183]. Upon activation, LDH can release their target mRNAs, allowing mRNA translation and cytokine production [183].

Aerobic glycolysis is also irreplaceable in innate immune cells because the innate immune cells require abundant energy sources to exert their anti-tumor functions. Such as NK cells rely on glycolysis to kill tumor cells [192, 193]. The number, viability, and cytotoxicity of NK cells in lung cancer are restrained due to an increased FBP1 expression, a gluconeogenesis-related enzyme that facilitate gluconeogenesis and inhibit glycolysis [192]. Some studies have indicated that inhibition of FBP1 significantly restored the glycolytic activity in NK cells and enhanced their cytotoxicity and cytokine-induced activation [192]. Other cells, including inflammatory/M1-like macrophages and dendritic cells (DCs) also experience a metabolic switch to a more glycolytic phenotype upon activation [194–196].

Warburg effect in tumor and non-tumor cells leads to an increased lactate production which is exported to the extracellular environment, causing high acidity in TME. This causes intracellular acidosis and suppresses the anti-tumor function of immune cells, leading to the loss of immune surveillance and progression of multiple kinds of cancer, including colorectal cancer [56]. Lactate had an inhibitory effect on CD8 + T cells [197]. Murine CD8 + T cells cultured in acidic media could uptake lactate, which leads to intracellular acidification [197]. The intracellular acidification can further inhibit the upregulation of NFAT, an essential transcription factor during CD8 + T cell activation and reduce the production of IFN-γ [197]. Proteomic analysis revealed that the protein levels of most glycolytic enzymes exert negative correlation with CD8 + T cell infiltration in deficient mismatch repair (dMMR)/microsatellite instability-high (MSI-H) CRC [198]. Further, MSI-H CRC samples with a higher glycolytic activity tend to be infiltrated with a less amount of CD8 + T cells, suggesting that glycolytic activity could be helpful to guide the clinical application of immune checkpoint inhibitor (ICI) or predict the outcomes of CRC patients who are receiving immunotherapy [198]. Also, the immune cells can differentiate into a more immunosuppressive phenotype, such as Tregs and M2-like macrophages to adapt to the harsh environmental conditions [199]. Moreover, lactate from CRC cells inhibits the phagocytic ability of TAMs by inducing the Ap-2α/Elk-1 axis which elevates the protein level of Sirpα, an immune checkpoint that negatively regulates the anti-tumor function of TAM [200]. On the contrary, the downregulation of lactate decreases the number of tumor-infiltrating Treg and myeloid-derived suppressor cells in CRC, thus improving the efficiency of immune therapy [201]. Further, lactate could also function as an agonist for the G-protein-coupled receptor 81 (GPR81) to deliver cell signaling. Lactate can activate GPR81 in cancer cells which leads to PD-L1 upregulation and tumor evasion [202]. Similarly, tumor-derived lactate can also induce activation of GPR81 in immune cells and activation of GPR81 in dendritic cells suppressed cell-surface presentation of MHCII and decreased the production of cAMP, IL-6, and IL-12 [203]. It has also been reported that GRP81 signaling induction impairs the pro-inflammatory ability of TAMs and activates the immunosuppression of myeloid-derived suppressor cells [204, 205]. Emerging evidence has proved the unique role of lactylation in regulating immune cells' function under hypoxia and acidic environment [206]. In CRC, the lactate accumulation in the tumor microenvironment can induce the METTL3 expression in tumor-infiltrating myeloid cells (TIMs) via histone lactylation [207]. METTL3, a m6A “writer’’, can activate the JAK/STAT pathway which leads to immunosuppression of TME and tumor progression [207]. The discovery of histone lactylation has provided new insights into the metabolic regulation of transcription and will expand our horizons on cancer treatment. In summary, these results have indicated that a higher concentration of lactate plays an important role in remodeling immunosuppressive tumor microenvironment.

It is important to note that the tumor microenvironment is distinct from the metabolic immune microenvironment under physiological conditions. As suggested by Otto H Warburg, highly proliferative tumor cells and disordered vascular cause a decrease in intratumoral glucose concentration, resulting in a competitive environment between immune and cancer cells. Under glucose deprivation, the phosphorylation of p70S6 kinase and eIF4E binding protein 1 were reduced, which could inhibit the synthesis of IFN-γ and impair the anti-tumor function of effector CD8 + T cells [208]. The microarray analysis further discovered that glucose restriction leads to the decrease of IFN-γ, GM-CSF, Perforin, Granzyme C and Cyclin D2, indicating that the anti-tumor function of CD8 + T cell was significantly impaired in a glucose-deprived environment [209]. Glucose deprivation in tumor cells attenuates the glycolytic ability of T cells, leading to inhibition of the mTOR pathway [210]. mTOR pathway plays an important role in the regulation of effector and regulatory T cell lineage commitment [211]. T cells with impaired mTOR activity could not differentiate into Th1, Th2, Th17 cells and exhibited high activity of Smad3, leading to the differentiation into Treg cells [211]. Furthermore, the low flux of glycolysis also impairs immunosurveillance by inhibiting the production of metabolic intermediates. A low concentration of glucose (0.1 mM) causes less production of phosphoenolpyruvate/PEP, a glycolytic metabolite that plays an important role in activating TCR-dependent Ca^2+^-NFAT signaling in anti-tumor CD4^+^ cells [212]. An absence of aerobic glycolysis promotes PD-1 expression and GAPDH binding to the 3’UTR of IFN-γ mRNA to negatively regulate the IFN-γ protein level in CD4 T Cells [191]. By contrast, Treg cells do not rely on a high dose of glucose and can be induced by AMPK signaling to exert their immune-suppressive functions [213]. These findings suggest that immune cell exhaustion can be overcome by reversing glucose restriction and creating a nutritionally adequate environment. However, a recent study by W Kimryn Rathmell et al. used PET tracers and measured the access glucose and glutamine uptake of specific cell subsets cultured in a metabolites-available environment [214]. The study found that TAMs and M-MDSCs exhibited significantly higher glucose uptake ability than cancer cells and T cells in the TME and maintained a robust glucose metabolism [214]. Inhibition of glutamine metabolism could enhance the glucose uptake of immune cells and cancer cells, suggesting that tumor-intrinsic physiological mechanism could regulate the glucose uptake of immune cells [214]. While glucose restriction may exist, a high concentration of glucose can be found in some kinds of tumors where exists tumor-infiltrating lymphocytes with impaired anti-tumor functions [215]. Therefore, immune exhaustion is not the only restriction of glucose but glucose as an energy resource to immune cells which is necessary for the antitumor immunity. However, other nutrients, metabolites, and changes in cancer cell physiology are also indispensable factors to construct the heterogeneous tumor microenvironment.

### Warburg effect connects carcinoma-associated fibroblasts and cancer cells

Cancer cells and carcinoma-associated fibroblasts (CAFs) have reciprocal metabolic interactions [216]. CAFs could prompt CRC cells to change their metabolism toward a more glycolytic phenotype [217, 218]. It has been observed that while co-culturing CRC cell line DLD1 with CAFs, the glucose uptake and lactate secretion rates were significantly strengthened while the glutamine anabolism and TCA cycle were restrained [217]. Similarly, another study verified that CAFs can enhance the 18F-FDG uptake and expression of GLUT1 and HK2 in CRC cells [218]. In addition, clinical CRC samples with a higher density of CAF tend to exhibit higher 18F-FDG uptake and are relevant to poor prognosis [218]. In turn, tumor cells also promote glycolysis in CAFs [218]. Both platelet-derived growth factor/PDGF and culture media of human CRC cell line HCT116 could induce the formation of CAFs. In addition, culture media-induced CAFs show a higher expression of HK2 and GLUT1 at both protein and transcription levels than PDGF-induced CAFs. Furthermore, the mRNA level of HIF1α under normoxia was also significantly enhanced in culture media-induced CAFs, indicating that tumor cells could significantly enhance the glycolytic rate of CAFs which might be achieved through a complex regulatory network [218].

It has been demonstrated that metabolic heterogeneity exists in tumors with different metabolic phenotypes such as aerobic glycolysis and OXPHOS in a certain type of cell [219, 220]. A portion of cancer cells and CAFs show a glycolytic phenotype, while other cancer cells utilize lactate as an energy source [219]. In detail, glycolytic CAFs could upregulate MCT4 which is a lactate efflux transporter and promote the secretion of lactate from CAFs to extracellular fluid while a part of tumor cells can uptake lactate and display oxidative metabolism [221]. This suggests that an intercellular metabolic circuitry, termed “the reverse Warburg effect,” exists in the tumor microenvironment which further enhances the metabolic plasticity of tumor cells. Such a cellular metabolic interaction allows tumors to react to changes in nutrient availability, thus maximizing cellular proliferation and growth.

### Exosome: an important vesicle for cell communication

Exosomes are nano-sized extracellular vesicles that contain a variety of bioactive molecules, such as nucleic acids, proteins, lipids, and metabolites and show striking potential to aid tumor diagnosis and treatment [222, 223]. Cells in the TME can secrete exosomes to interact with and dynamically remodel the metabolism of other cells [15]. For example, tumor-derived exosomes can increase glucose uptake and inhibits mitochondrial oxidative phosphorylation in macrophages which reprogram macrophages into glycolytic-dominant metabolism and immunosuppressive phenotype [224]. Further, the non-tumor cells can regulate the glycolytic metabolism of cancer cells and a growing body of evidence indicates that a complex exosome-mediated cell-to-cell communication exists in the tumor microenvironment of CRC [15, 225]. The exosome-associated proteomics of CRC cell lines identified that cancer-derived EVs are specifically enriched in glycolytic pathways and lead to a metabolic switch in CAFs that undergo aerobic glycolysis compared to normal fibroblasts [226]. Reciprocally, exosomes secreted by CAFs can inhibit mitochondrial oxidative phosphorylation and increase glycolysis in cancer cells [227]. It has also been demonstrated that non-coding RNA is an important messenger to regulate the glycolytic activity of distant cells. For instance, circular RNA hsa_circ_0005963 (ciRS-122) in oxaliplatin-resistant CRC cells can be packaged into exosomes and transported into oxaliplatin-sensitive CRC cells to promote glycolysis and drug resistance through miR-122 sponging and PKM2 upregulation in vitro and in vivo [228]. Furthermore, the exosome is an important mediator between primary tumor and premetastatic niche which promotes metastasis and is one of the mechanisms to reprogram recipient cells' metabolism. However, exosomal miR-122 was able to increase nutrient availability in the premetastatic niche by inhibiting the glucose utilization of distant organs, thereby meeting the high energy demand and low ATP-generating efficiency of cancer cells [229].

## Warburg effect promotes drug resistance in CRC

Despite the application of therapeutics including chemotherapy, targeted therapy and immune therapy has markedly improved patients’ survival, a large number of patients still undergo disease progression due to drug resistance [7, 230]. The mechanisms of drug resistance include the increasing drug efflux, drug inactivation, alterations in drug targets, enhanced DNA damage repair, evasion of cell death, epigenetic modifications, activation of survival signaling, cancer stemness, and EMT [231]. Emerging evidence has indicated that increasing the Warburg effect in the tumor cells could enhance CRC cell resistance to anti-tumor drugs and the tumors that were more resistant to anti-tumor drugs tend to be more glycolytic [232, 233].

### Chemotherapy

Although therapeutics based on 5-fluorouracil with oxaliplatin or irinotecan markedly kill CRC cells, a proportion of tumor cells still survive, leading to drug resistance and disease progression [7]. Glycolytic metabolism contributes to the resistance to chemotherapy. Research indicated that a higher expression of glucose transporters such as GLUT1, GLUT4, and SGLT1 was found in colorectal cancer from non-responsive patients than those responsive to 5-FU. Upregulation of glucose transporters can enhance aerobic glycolysis to produce more pyruvate to confront ROS-induced necroptosis and to regulate cell cycle into a quiescent state [234, 235]. Another study also found that high glucose attenuates antiproliferative effect and cytotoxicity of 5-Fluorouracil in human colon cancer cells [236]. As a hallmark of the Warburg effect, the acidic tumor microenvironment has strong adverse effects on cancer treatment. A study indicated that the inhibition of the lactate efflux sensitizes tumors cells to cisplatin treatment [237]. As suggested, the Warburg effect also supplies a considerable amount of energy to tumor cells. Tumor cells with highly glycolytic metabolism could generate sufficient ATP to support the efflux function of the ATP-binding cassette (ABC) family and create an acidic microenvironment, promoting drug efflux pumps activity [139, 238]. In 5-FU resistant CRC cell lines, the expression of drug efflux transporters, especially the ABC transporter family was observed to be upregulated which reduced the intracellular availability of anti-tumor drugs [239]. It has also been revealed that the Warburg effect can also resist the induction of cell death by inhibiting apoptosis [240]. Enhanced glycolytic metabolism could decrease the intracellular level of ROS which is regarded as an important factor inducing apoptosis [241, 242]. Glycolytic enzymes could also rescue ROS-induced apoptosis by directly regulating the process of apoptosis [243]. For example, PKM2 has been reported to translocate to mitochondria and phosphorylate Bcl2, which increases the Bcl2 expression to inhibit apoptosis [243]. Similarly, HK2 could also translocate to mitochondria and protect tumor cells from apoptosis [244]. Conversely, inhibition of glycolysis in the human CRC cell line leads to increased apoptosis rates and decreased resistance to 5-Fu [245]. The decreased aerobic glycolysis always accompanies relevant upregulation of apoptosis. Therefore, while measuring cancer cell ability to resist drugs, the detection of degree of apoptosis are widely considered [246]. Multiple findings have demonstrated the existence of glycolysis-induced drug resistance in CRC. However, the role of other mechanisms such as DNA damage repair, EMT and stemness to induce resistance are largely unknown in CRC. Hence, more in-depth studies are needed to unravel the detailed mechanisms and hub genes in drug resistance, which would help us find potential therapeutic targets to overcome drug resistance in CRC.

### Anti-angiogenic therapy

Anti-angiogenic treatment has become an attractive therapeutic avenue in colorectal cancer [247]. Bevacizumab, an anti-VEGF monoclonal antibody, was the first biologic agent approved for metastatic colorectal cancer and the addition of bevacizumab to other chemotherapy backbones has been shown to better progression-free survival [7]. However, resistance to anti-VEGF therapy has been observed and metabolic adaptation plays a vital role in this progression [248, 249]. Bevacizumab-resistant cells exert higher glucose uptake ability and glycolytic activity than bevacizumab-responsive tumor cells [250]. In addition, Bevacizumab treatment leads to a more hypoxic environment, where tumor cells change their metabolic phenotype toward a more glycolytic one [251]. Upregulation of HIF-1a can promote neovascularization by promoting tumor cells to produce pro-angiogenic factors such as PDGF-B, FGF-2, VEGF-A, VEGFR1 and angiopoietins [252]. Hence, highly glycolytic metabolism can be considered as a metabolic feature regarding the resistance to Bevacizumab [249, 253]. Furthermore, Xu et al. found that CRC cells refractory to bevacizumab treatment not only undergo hyperactive glycolytic phenotype, but also occur persistent impairment of mitochondria [254]. Therefore, targeting glycolysis has a promising therapeutic potential in bevacizumab-resistant patients because the impaired mitochondria in bevacizumab-resistant cells makes them more dependent on glycolytic metabolism. In CRC, treatment of bevacizumab-resistant cells with the HK2 inhibitor 3-BrPA caused cell senescence in vitro and smaller tumor volume and longer survival in vivo [254].

### Immune checkpoint inhibitors (ICIs)

Immune checkpoint inhibitors (ICIs) such as nivolumab or pembrolizumab has been approved to treat colorectal cancer and provided durable responses and disease control in patients with MSI-H tumors [7]. However, the majority of patients do not benefit from immunotherapy, which might be correlated with the immunosuppressive TME. The activation and anti-tumor function of effector T cells rely on sufficient nutrients. However, glucose deprivation limits T cell persistence and function [255]. In addition, immunosuppressive metabolic byproducts such as lactic acid can significantly abolish the T cell mediated lysis of cancer cells [255]. A recent study found that acidic TME can inhibit the expression of PD-1 in effector T cells while induce the expression of PD-1 in Treg cells because Treg cells can utilize lactate to promote the nuclear translocation of NFAT1 and further activate the transcription of PD-1 [16]. Therefore, PD-1 blockade could increase the activity of PD-1 + Treg cells, resulting in the resistance to immunotherapy [16]. Transcription analysis also revealed a negative correlation between tumor glycolysis activity and tumor-infiltrated CD8 + T cells [198]. Additionally, elevated level of LDH in patient serum samples predicts poor therapeutic responses to the anti-PD-1 antibody pembrolizumab [256, 257]. Hence, targeting glycolytic metabolism is a potential therapeutic method to attenuate the immunosuppressive microenvironment and overcome the resistance to ICIs.

## Glycolytic metabolism is a therapeutic target in CRC

**Fig. 4:**
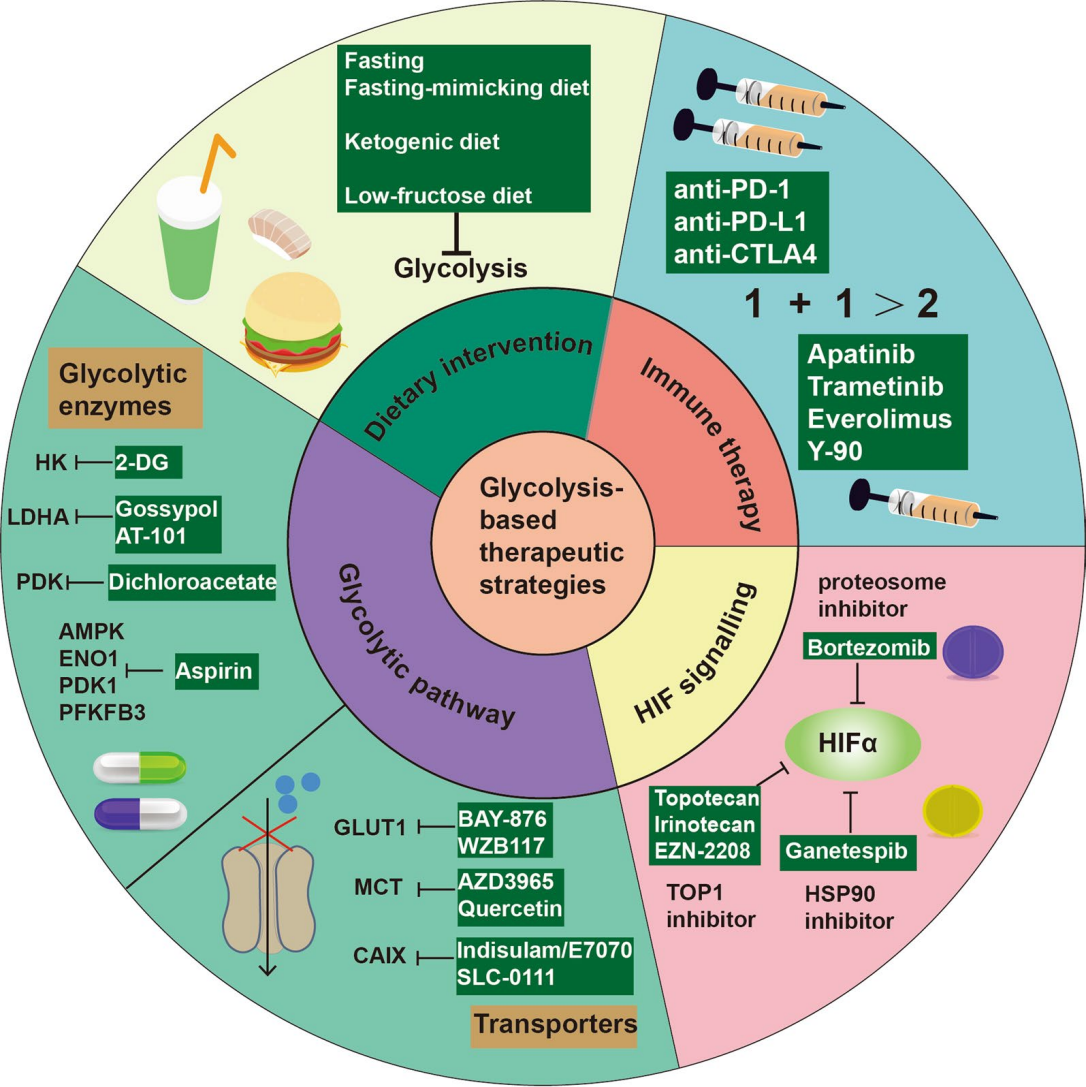
Summary of glycolysis-based therapeutic strategies for CRC. Arrows indicate positive modulations or transitions, while blunt ends indicate negative modulations

The metabolic differences exist between the tumor and non-tumor cells, suggesting targeting glycolytic metabolism as a promising and tempting method in anticancer strategy (Fig. 4). Several pharmaceutical companies have developed a series of small-molecule inhibitors or compounds that inhibit glycolysis and some of which have been used in clinical trials. In CRC, some compounds have been reported to exert an anti-tumor effect in a series of preclinical studies, highlighting the translation of these findings into clinical trials (Table 1). In addition, dietary intervention in cancer treatment has promising effects, especially in diet-related CRC. The success of preclinical studies of small-molecule inhibitors and dietary intervention in cancer treatment promotes their clinical application (Table 2).

**Table 1:** Small-molecule inhibitors targeting glycolytic metabolism in CRC

Compound	Target	Combined therapy	Outcomes	Reference
*BAY-876*	GLUT1	NA	Inhibit proliferation	[258]
*WZB117*	GLUT1	5-Fu	Reduce 5-Fu resistance	[261]
*2-DG*	HK	5-Fu/Oxaliplatin	Increase chemosensitivity, inhibit migration	[265]
*Gossypol*	LDHA	5-Fu	Increase chemosensitivity, inhibit tumor growth in vivo and vitro	[270–272]
*Dichloroacetate*	PDK	5-Fu/Oxaliplatin	Increase chemosensitivity, inhibit tumor growth in vivo and vitro	[273–275]
*AZD3965*	MCT1	NA	Inhibit growth and glycolysis	[276]
*SLC-0111*	CAIX	5-FU	Increase chemosensitivity, inhibit tumor growth	[277, 278]

**Table 2:** Clinical trials that target glycolytic metabolism in CRC

Compound	Target	Phases	Combined therapy	Status	Reference
*Aspirin*	CRLM resection	Phase II/III	NA	Recruiting	NCT00490139
*Aspirin*	MSI-H/dMMR or TMB-H	Phase II	PD-1 antibody	Recruiting	NCT03638297
*EZN-2208*	Advanced CRC	Phase II	± Cetuximab	Unknown	NCT00931840
*Quercetin*	CRC	Not Applicable	Sulindac, Curcumin, Rutin	Terminated	NCT00003365
*Indisulam*	mCRC	Phase II	Capecitabine/Irinotecan	Completed	NCT0016585/NCT00165867
*FMD*	CRC	Not Applicable	Unknown	Completed	NCT03595540 NCT03340935
*Fasting*	CRC	Not Applicable	Chemotherapy	Enrolling by invitation	NCT04247464
*Ketogenic diet*	CRC	Not Applicable	Radiotherapy	Completed	NCT02516501

### Targeting glycolytic enzymes or transporters: small-molecule inhibitors at the forefront of targeted drug discovery

#### GLUT1 inhibitor

Glycolysis is initialized by the GLUT-mediated glucose intake and the expression of GLUT1 is higher in the TCGA CRC cohort than in normal cells [258]. Small-molecule inhibitors targeting GLUT1 have been found to exert potent anti-tumor functions in several cancers [259, 260]. It has been revealed that WZB117 is a GLUT1 inhibitor that has been reported to reduce the cell viability of the 5-Fu-resistant CRC cell line [261]. BAY-876 is another inhibitor that specifically targets GLUT1 and can significantly inhibit the growth of CRC in both in vitro and in vivo experiments [258]. In addition, combined treatment using BAY-876 and DBI-1, an electron transport chain inhibitor, showed a more significant inhibitory effect, suggesting that metabolic plasticity exists in CRC and GLUT1-induced glucose uptake and mainly involves glycolytic metabolism [258].

#### HK2 inhibitor

2-Deoxy-d-glucose (2DG) is a glucose analog that can be taken up into cells and phosphorylated by HK to form 2-DG-6-phosphate [262, 263]. 2-DG-6-phosphate cannot be transformed into fructose-6-P which leads to the accumulation of 2-DG-6-phosphate and subsequent inhibition of HK [262]. 2DG has been proved to exert anti-tumor functions and synergistically work with small-molecule compounds or chemotherapy in multiple cancers [262]. In addition, combined therapy that includes 2DG has entered clinical trials (NCT00096707, NCT00633087). To date, the clinical trial of 2DG in CRC focuses on cancer diagnosis in which the radiolabeled 2DG measures glucose uptake throughout the body, whereas their pharmacological effect is only tested in preclinical studies [264]. In the CRC cell line, a preclinical trial indicated that 2-DG significantly inhibited glycolysis, reversed EMT, reduced migration ability, increased apoptosis, and promoted the chemosensitivity to 5-fluorouracil and oxaliplatin [265]. An outstanding anti-tumor performance was observed when 2-DG was used in combination with daunorubicin or alpha-tocopheryl succinate to treat CRC cell line, indicating 2-DG is a potent adjuvant agent for chemotherapeutic agents used and can be used clinical studies [266, 267].

#### Gossypol

Gossypol is a natural compound derived from the cotton plant, while AT-101 is a gossypol ( −)-enantiomer which shows a slower degradation rate than gossypol and therefore is more biologically active [268]. Gossypol/AT-101 could significantly inhibit proliferation and promoted apoptosis in multiple cancers types [268]. Gossypol has also been reported to reduce glycolysis by inhibiting LDHA [269]. It was observed that after Gossypol treatment, the cell viability of COLO225 which is a human colon cancer line, was significantly reduced along with downregulation of HIF1A, GLUT1, and GAPDH, indicating that Gossypol could inhibit tumor growth partly through glycolytic pathways [270]. Another study reported that gossypol could inhibit the growth of xenograft tumors in mice by promoting the production of ROS and the upregulation of ROS may derive from the inhibition of glycolytic metabolism [271]. In CRC, the combined therapy using 5-FU and gossypol exerts a better effect than using 5-FU alone, suggesting the anti-tumor function of Gossypol which makes it a potential candidate to be used in combination with other drugs and increase treatment efficacy [272]. The potent anti-cancer effect of Gossypol/AT-101 in preclinical studies have led the use of AT-101 in clinical trials. Clinical studies to test the effect of AT-101 in the human body have been carried out in multiple kinds of cancers, such as non-small cell lung cancer (NCT01977209), glioblastoma (NCT00540722), prostate cancer (NCT00666666), head and neck squamous cell carcinoma (NCT01285635).

#### Dichloroacetate

Dichloroacetate (DCA) is a structural analog of pyruvate and can inhibit pyruvate dehydrogenase kinase (PDK), which finally reverses cancer glycolytic metabolism and promotes apoptosis [279]. Several studies mostly in brain cancer have described the anti-tumor function of DCA in vivo and in vitro. Among the 9 clinical trials that are registered on clinicaltrials.gov, 5 are in phase I trials which tested the clinical pharmacology and toxicity of DCA. A randomized, placebo-controlled, double-blind phase II study (NCT01386632) tested whether DCA can be an adjuvant drug and enhance the effect of cisplatin-based chemoradiotherapy (CRT) in locally-advanced HNSCC and found that DCA group showed higher response rates, lower pyruvate and lactate level than placebo control and no significant differences in grade 3/4 adverse event rates [280].

However, there have been no clinical trials of DCA in CRC. Meanwhile, a preclinical study suggested that the treatment of CRC cell line with DCA (20 mM) downregulates glycolysis and promotes OXPHOS to inhibit the growth of CRC cells with no inhibitory effect on non-cancerous cells [273]. Another study demonstrated that DCA treatment predominantly promotes the anti-tumor effect of fluorouracil and oxaliplatin in CRC cell lines [274, 275]. In 2016, a case study reported that a patient with CRLM gained long-term stabilization after using DCA as a cytostatic agent [281]. The patient experienced serious side effects from FOLFIRI + bevacizumab treatment and chose to take DCA regularly without any other active chemotherapy from 2013 to 2016. Surprisingly, the patient experienced four years of disease stability with no serious side effects [281]. This case suggested that DCA has immense potential in CRC treatment and further clinical investigation should be carried out to explore the functions of DCA.

#### Aspirin

Aspirin is a well-known oral drug widely used as an analgesic and antipyretic drug. Moreover, it has been applied to prevent and treat cancer [282]. The classical anti-cancer mechanism of aspirin is to inhibit the COX enzymes activity and inflammation [282]. In addition, aspirin could inhibit cancer progression by reducing glycolytic levels in cancers [283]. Salicylate is the metabolite of aspirin which could activate AMPK (5’adenosine monophosphate-activated protein kinase) signaling which further inhibits mTOR signaling and suppress energy metabolism, including glycolysis [284]. It has been reported that aspirin directly downregulates the level of ENO1, PDK1, and PFKFB3 to attenuate glycolysis and tumor progression [285–287]. In CRC, multiple preclinical studies explored the potential anti-tumor mechanisms of aspirin. A recent study found that aspirin interacted with p300 and promoted the apoptosis of colorectal cancer stem cells through p300-AcH3K9-FasL axis [288]. The combined use of aspirin and 5-Fu significantly enhanced the inhibitory function of 5-Fu on the CRC cell line and xenograft CRC model [289]. Interestingly, aspirin had a better therapeutic effect on PIK3CA mutant CRC [290]. While PIK3CA mutant cells mainly rely on PI3K/Akt/mTOR pathway to sustain rapid proliferation and active metabolic activity and aspirin could notably suppress this pathway, leading to excellent inhibitory effects [290].

Multiple clinical studies have demonstrated the potent effect of aspirin in the prevention of CRC [282]. A double-blinded, randomized, placebo-controlled clinical trial of Asian patients with colorectal adenomas and adenocarcinomas indicated that the use of aspirin (100 mg/day for 2 years) significantly reduced colorectal tumor recurrence (OR = 0.6) [291]. Similarly, another clinical trial revealed that daily use of aspirin in CRC patients can reduce the recurrence of adenoma [292]. Long-term intake of 600 mg aspirin per day also substantially inhibited the occurrence of cancer in patients with Lynch syndrome [293]. However, some trials showed no significant effect of aspirin on CRC patient treatment, indicating the genetic differences in the patients, thus highlighting that the application of aspirin needs precise identification of individuals [294, 295]. To date, a total of 41 clinical trials using aspirin in CRC are listed at https://www.clinicaltrials.gov/ and the majority of clinical trials focus on identifying the effect of aspirin in the prevention of CRC and the risk category that are most likely to benefit from the use of aspirin. For example, a study of phase II/III ASAC trial of aspirin/acetylsalicylic acid treatment in patients with resection for CRC liver metastases evaluated whether low-dose aspirin can improve disease-free survival in these patients (NCT03326791). Notably, a phase II clinical trial that aims to evaluate the efficacy and safety of the combination of PD-1 antibody and COX inhibitor in CRC patients with MSI-H/dMMR or high tumor mutation burden is now recruited in Guangzhou, China (NCT03638297). Thus, the new wine of immune therapy in the old bottle of aspirin will further deepen our understanding for this drug.

#### MCT inhibitor

AZD3965 is a small-molecule inhibitor that specifically targets MCT1. In MCT4 deficient cell line LS174T, AZD3965 significantly decreased intracellular pH level and inhibited glycolysis [276]. In addition, a phase I clinical trial evaluated the safe dose of AZD3965 in the advanced tumor and was completed in the UK (NCT01791595), indicating that the 20 mg oral dose of AZD3965 was generally tolerated by patients [296]. Quercetin, a natural compound, is also known as an anti-tumor drug that found to inhibit the occurrence and development of tumors by various mechanisms [297]. Also, quercetin has been regarded as a non-specific MCT inhibitor [298]. Quercetin can significantly inhibit proliferation, promote cell death and reduce glycolytic activity in the CRC cell line [299]. In addition, quercetin enhances the cytotoxicity of 5-FU by inhibiting the lactate transport in CRC cells [299]. Quercetin is used as a chemosensitizing agent for kidney cancer (NCT02446795), pancreatic ductal adenocarcinoma (NCT01879878), and chemopreventive drug in prostate cancer (NCT01538316), colorectal cancer (NCT00003365) and oral carcinogenesis (NCT01961869, NCT03476330). A clinical trial of curcumin and quercetin treatment in 5 familial adenomatous polyposis (FAP) patients with prior colectomy showed that treatment with curcumin and quercetin for a half year decreased the number of polyps by 60.4% and the size of polyps by 50.9% [300]. This study further highlighted the therapeutic effect of quercetin in CRC and laid the foundation for subsequent clinical trials in CRC treatment.

#### Carbonic anhydrase

Carbonic anhydrases (CAs) catalyze a reversible reaction in which CO_2_ and water are transformed into intracellular bicarbonate and a proton is released extracellularly [301]. Tumor cells with a high glycolysis rate produce plentiful lactate which exists in the form of lactate– (Lac–) and H^+^. Tumor cells rely on MCTs as well as CAs to export redundant protons and lactate and maintain the acid–base balance [302]. In addition, CAIX has been reported to significantly promote the lactate flux of MCT1 and glycolytic rates in a non-catalytic manner [303, 304]. Indisulam/E7070 is an efficient inhibitor against multiple CAs [305] and could not only suppress the proliferation of CRC cell lines but also lead to a decrease in the tumor volume of xenograft CRC models, highlighting the powerful anti-tumor ability [306]. Further, two phase II clinical trials tested indisulam as an adjuvant drug and observed the enhanced therapeutic effects of capecitabine (NCT00165854) or irinotecan (NCT00165867) which were carried out in metastatic CRC patients. SLC-0111 is another compound that specifically targets CAIX and is currently under clinical trials. In CRC, SLC-0111 could promote apoptosis and necrosis of CRC cell lines along with enhanced response of 5-FU to chemotherapy in CRC [277, 278]. Generally, CAs play an important role in maintaining a high level of glycolysis in CRC and can be potential metabolic targets in cancer therapy.

#### HIF-1

During the past several years, identifying HIF-1 inhibitors has become a novel strategy to treat CRC and many drugs are being evaluated in clinical trials due to the central role of HIF-1 in maintaining high glycolysis rates [307]. Ganetespib, an HSP90 inhibitor, has been reported to reduce HIF­1α stability and bortezomib, a proteasome inhibitor, can inhibit HIF­1α transactivation to further inhibit glycolytic metabolism [308]. In preclinical studies, topoisomerase 1 (TOP1) can promote the translation of HIF­1α and is targeted by TOP1 inhibitors, such as topotecan and irinotecan/CPT-11 [302, 309]. Their anti-tumor function has been widely accepted and is being studied in multiple clinical trials. However, whether their anti-tumor functions are mediated by the inhibition of HIF­1α remains largely unknown [302]. EZN-2208 is a derivant of SN38 which is an active part of irinotecan and strongly suppresses the expression of HIF-1α/HIF-2α and HIF-induced downstream targets, such as GLUT1 [310, 311]. EZN-2208 shows more powerful, sustained HIF-1α inhibition compared to CPT-11, thus becoming an ideal HIF-1α inhibitor [311]. A phase I clinical trial (NCT01251926) of EZN-2208 treatment in patients with refractory solid tumors showed that 5 of 7 patients showed a decrease in HIF1A [312]. Moreover, the effect of EZN-2208 was tested in a randomized Phase II trial (NCT00931840) that enrolled a total of 213 patients with advanced CRC [313]. However, EZN-2208 did not induce objective radiographic responses in KRAS-mutant patients who were resistant to CPT-11. Meanwhile, similar OS and PFS were discovered between cetuximab + EZN-2208 and cetuximab + CPT-11 group and these results may be the result of unfavorable pharmacokinetics of EZN-2208 [313].

### Dietary intervention- a focus on tumor glycolysis

In the past decade, the role of diet has caught the attention of scientists because metabolites and biomolecules in the blood can significantly influence tumor growth, especially in CRC [314]. Dietary intervention is cheap, available, and could synergize with standard therapy to enhance the therapeutic effect [315]. Understanding the importance and practicability of dietary intervention and its rational application will help us understand and gain better insights into cancer treatment. Some reviews have discussed the dietary approaches that influence cancer therapy and in this review, we mainly focus on the role of dietary intervention in inhibiting CRC glycolytic metabolism [316, 317].

Fasting or fasting-mimicking diet (FMD) can lead to chronic calorie restriction and reduced levels of blood glucose, insulin, and insulin-like growth factors -1 (IGF-1), which further inhibits insulin-mediated PI3K/AKT/mTOR pathway and aerobic glycolysis [318]. A recent report has found that fasting could induce the upregulation of FDFT1, a tumor suppressor gene to attenuate the AKT/mTOR/HIF pathway, thus inhibiting tumor glycolysis and proliferation in a CRC mouse model [319]. A single-arm, phase I/II clinical study (NCT03595540) evaluated the safety and feasibility of 5-day FMD in malignant tumors, including CRC, indicating the combination of FMD and chemotherapy is safer and feasible with a reduction of serum IGF1 and IGFBP3 [320]. In another clinical trial (NCT03340935), fasting-mimicking diet reduced blood glucose and growth factor concentration with reliable safety. [321]. Recently, a pilot clinical study (NCT04247464) is undergoing in Madrid, Spain to test whether short-term fasting (24 h before and 24 h after chemotherapy) can improve anti-tumor effect and reduce chemotherapy toxicity. The ketogenic diet is another method to limit glycolytic metabolism by reducing glucose levels in cancers [322]. Animal studies have proved that a ketogenic diet can inhibit tumor growth, laying a foundation for clinical trials [323, 324]. The KETOCOMP study (NCT02516501) intended to explore the impact of the ketogenic diet on CRC patients undergoing radiotherapy [325]. Compared to patients with a standard diet, patients with a ketogenic diet showed a better mental state, better physiological indicators, and a trend contributing synergistically to pathological tumor response, thus demonstrating the therapeutic potential of ketogenic diet in CRC treatment and the necessity of future confirmation in larger studies [326, 327].

Few epidemiological studies have indicated that a high-fructose diet is associated with tumorigenesis in CRC [317, 328]. APC mutant mice were raised with high-fructose corn syrup to investigate the mechanism of fructose-induced tumorigenesis in CRC [329]. The results indicated the presence of more tumor numbers and higher tumor grade in HFCS-treated mice compared to control group [329]. Furthermore, researchers have found that fructose can be transformed into fructose-1-phosphate and activate the glycolytic metabolism and fatty acid metabolism that promote tumor development [329]. Considering the role of fructose in fueling cancer glycolytic metabolism, diets that limit fructose uptake can be a potential intervention to inhibit tumor development.

### Targeting Warburg metabolism to improve the effect of ICIs

Immune checkpoint inhibitors (ICIs) dramatically changes the therapeutic outcome of metastatic tumors [330, 331]. In 2017, pembrolizumab (PD-1 inhibitor) was first approved by FDA to treat MSI-H CRC [332]. An important clinical trial in CRC immunotherapy is KEYNOTE-177 (NCT02563002) which is a randomized, phase III trial that uses pembrolizumab as first-line treatment to compare the efficiency with 5-FU-based traditional chemotherapy in MSI-H/dMMR CRC patients [333]. Pembrolizumab treatment leads to longer progression-free survival (PFS) than 5-FU-based chemotherapy, thus leading to the recommendation of the pembrolizumab as a first-line treatment option in MSI-H/dMMR mCRC in NCCN guidelines [333, 334]. Despite the immense progress of ICIs in MSI-H CRC, their application in microsatellite-stable (MSS) CRC is still limited. Therefore, the focus of clinical trials treating MSS CRC is a combination therapy using both ICIs and other drugs to transform “cold” tumors into “hot” tumors [335].

Warburg metabolism can significantly influence the TIME and have close interactions with the expression of immune checkpoints, suggesting glycolysis-based therapy with ICI could reverse the immunosuppressive environment, thus overcoming the resistance in single-agent ICI [76, 168]. Although the application of classical glycolysis inhibitors in conjunction with ICIs in clinical trials is largely unknown, the preclinical trials have demonstrated their therapeutic potential. For instance, treatment of mice bearing CT26 colorectal tumors with aspirin and anti-PD-1 reduced tumor growth and was followed by rapid and complete shrinkage in 30% of mice, whereas monotherapy showed little efficacy [336]. Another study demonstrated that the LDHA inhibition could inhibit tumor glycolysis and improve the efficacy of PD-1 blockade in a murine pMMR CRC model, which was consistent with the clinical findings that highlighted the negative correlation of anti-PD-1 therapeutic efficiency with the serum LDH levels in patients [256, 257, 337]. In addition, the PFKFB3 inhibitor PFK-158 has been reported to improve the therapeutic responses to antibodies against CTLA-4 in a mouse B16 melanoma model [338]. Moreover, some chemotherapy drugs such as apatinib (VEGF inhibitor), trametinib (MEK inhibitor) can inhibit glycolysis and provide new insight into the anti-tumor mechanisms [339, 340]. Their application in conjunction with ICIs may gain better therapeutic benefits in CRC (Table 3). Some studies have already reported the combination benefit of glycolysis-related drugs and ICIs in cancer treatment. According to the results from CAP 01 trial using combined therapy (anti-PD-1 plus apatinib) in 20 patients with chemorefractory gestational trophoblastic neoplasia, the objective response rate (ORR) was 55% with acceptable toxicity and 10 patients had a complete response. Similar trials are also undergoing in CRC, which may lead to breakthroughs in CRC immunotherapy [341]. Another phase I/II clinical trial using combined PD-1, BRAF and MEK inhibition indicated that spartalizumab (anti-PD-L1) plus dabrafenib and trametinib led to an ORR of 78%, including 44% complete responses (CRs), highlighting glycolysis-inhibiting trametinib is an important reagent for cancer therapy and an adjuvant candidate to immunotherapy [342].

**Table 3:** Clinical trials combining ICIs with chemotherapy/radiotherapy in CRC treatment

Compound	Target	Combined therapy	Potential metabolic pathway	Outcomes	Reference
*Camrelizumab (anti-PD-1)* + *Apatinib(VEGF inhibitor)*	Advanced CRC	PhaseI/II	Apatinib suppresses glycolysis by inhibiting GLUT4/glucose uptake [339, 344]	Recruiting	NCT04067986
*Camrelizumab (anti-PD-1)* + *Apatinib(VEGF inhibitor)*	dMMR/MSI-H CRC	Phase II		Recruiting	NCT04715633
*Durvalumab(anti-PD-L1)* + *Trametinib(MEK inhibitor)*	MSS mCRC	Phase II	Trametinib can inhibit glycolysis through PKM2/c-myc axis [340, 345], reduce F-FDG uptake [346], inhibit glycolytic level in BRAF melanoma cells [347]	Active, not recruiting	NCT03428126
*Nivolumab(anti-PD-1)* + *Trametinib (MEK inhibitor)* ± *Ipilimumab(anti-CTLA4)*	mCRC	PhaseI/II		Recruiting	NCT03377361
*PDR001(anti-PD-1)* + *Trametinib (MEK inhibitor)* + *Dabrafenib (Raf inhibitor)*	mCRC with BRAF V600E mutation	Phase II		Recruiting	NCT03668431
*PDR001(anti-PD-1)* + *Everolimus (mTOR inhibitor)*	*CRC*	Phase I	Everolimus inhibits mTOR-mediated glycolysis [348, 349]	Completed	NCT02890069
*Y-90(radioembolization)* + *SBRT* + *Durvalumab(anti-PD-L1)* ± *Tremelimumab(anti-CTLA4)*	CRLM	Phase I	Yttrium-90-radioembolization can inhibit tumor glycolysis and decrease TLG (total lesion glycolysis) measured by F-FDG PET-CT [350–352]	Withdrawn	NCT03802747
*Y-90(radioembolization)* + *Nivolumab(anti-PD-1)*	CRLM	PhaseI/II		Withdrawn	NCT03307603
*Y-90(radioembolization)* + *Durvalumab(anti-PD-L1)*	CRLM	PhaseI/II		Recruiting	NCT04108481

Is Warburg metabolism the Achilles Heel of TIME? It is important to notice the overlap in metabolic patterns between tumor and anti-tumor immune cells as the inhibitors targeting glycolysis may impair both tumor and antitumor immune cells [76]. For instance, 2-DG could significantly inhibit the proliferation, glucose consumption, and lactate production of both CD4 and CD8 T cells [343]. In addition, the IFN-γ, TNF,

IL-10, and IL-4 production were also inhibited upon 2-DG treatment in effector CD4 T cells [343]. In another study, significantly reduced ability to expand and induce inflammatory reaction can be observed in Teff with Glut1 deficiency [353]. However, Treg cells can proliferate and exert immunosuppressive function in a Glut1-independent manner [353]. Therefore, the subtle differences in metabolic inhibition of individual cells need to be identified and verified for metabolic vulnerability in cancer and immune cells.

### Advanced technologies identify personal metabolic profiles and guide precision treatment

Despite the fact that tumor cells are generally glycolytic, heterogeneous metabolic patterns or preferences still exist among different individuals with the same types of cancer and even in the same sample. Metabolic heterogeneity is important because it influences therapeutic vulnerabilities and may predict clinical outcomes. The application of advanced technologies such as single-cell sequencing and multi-omics analysis can help identify the metabolic similarities and differences among individuals in different tumors. Single-cell sequencing can help us find the metabolic vulnerabilities in certain cell type. For instance, single-cell RNA sequencing data of CRC patients discovered low activity of the MondoA–thioredoxin-interacting protein (TXNIP) axis in Tregs, which can upregulate their glycolytic level [354]. Depletion of the MondoA-TXNIP axis induced hyper-glycolytic Th17-like Tregs, which facilitated Th17 inflammation, promoted CD8 + T cell exhaustion, and drove colorectal carcinogenesis [354]. Using transcriptomic data and bioinformation analysis, a molecular subtype with high level of glycolytic activity can be identified in triple-negative breast cancer, which was characterized by worse prognosis and increased production of glycolytic metabolites [355]. Cancer cells which are clustered into glycolytic subtype showed higher sensitivity to glycolysis inhibitors [355]. Glycolysis has close connections with malignant phenotypes, suggesting that tumors with high glycolytic activity might be associated with worse prognosis and targeting glycolytic metabolism in these patients could receive more therapeutic beneficials. From the perspective of this review, further studies should be focused on exploring the metabolic heterogeneity in CRC. Also, the metabolic subtype of CRC is also worthy to explore because this helps to find a group of patients that are sensitive to metabolic inhibitors. In addition, the majority of the CRC are not applicable to immune therapy. Single-cell sequencing can help us learn about the metabolic feature of immune cells and find metabolic targets to heat the “cold” tumor microenvironment.

## Conclusions

Cancer metabolism fuels and drives cancer development. Warburg effect is the earliest metabolic feature that is found in tumors and is continuously evolving giving newer insights to cure cancer. In this review, we summarized the crosstalk between Warburg metabolism and CRC, highlighting the irreplaceable role of glycolysis in promoting CRLM and remodeling the tumor microenvironment. The extensive regulation of glycolysis in CRC development makes it a potential therapeutic target. Along with the development of classical small-molecule inhibitors, dietary intervention studies are increasing the survival rates of cancer patients and emerging as a new field of study. The rapid rise of immunotherapy promotes the development of immunometabolism and emerging evidence has proved the potential of targeting glycolysis and enhancing immunotherapy efficiency. CRLM and immunosuppression in MSS CRC are two challenges in CRC treatment. Thus, future drug design should focus on maximizing tumor and immunosuppressive cell inhibition while try avoiding damage to normal and anti-tumor immune cells. Further, it is also important to encourage medical organizations to perform clinical studies based on glycolysis.

## Data Availability

Not applicable.

## Abbreviations

2DG: 2-Deoxy-d-glucose
2PG: 2-Phosphoglycerate
3PG: 3-Phosphoglycerate
ABC: ATP-binding cassette
ALDH: Aldehyde dehydrogenase
AMPK: 5’Adenosine monophosphate-activated protein kinase
bFGF: Basic fibroblast growth factor
CAFs: Carcinoma-associated fibroblasts
CAIX: Carbonic anhydrase IX
CAXII: Carbonic anhydrase XII
CRC: Colorectal cancer
CRLM: Colorectal cancer liver metastasis
CRs: Complete responses
CRT: Chemoradiotherapy
CSCs: Cancer stem cells
DCA: Dichloroacetate
DCs: Dendritic cells
dMMR: Deficient mismatch repair
ECM: Extracellular matrix
EMT: Epithelial-to-mesenchymal transition
F1,6BP: Fructose-1,6-biphosphate
F2,6BP: Fructose-2,6-bisphosphate
F6P: Fructose-6-phosphate
FAP: Familial adenomatous polyposis
FMD: Fasting-mimicking diet
G1,3DP: Glycerate-1,3-diphosphate
G3P: Glyceraldehyde-3-phosphate
G6P: Glucose-6-phosphate
GAPDH: Glyceraldehyde-3-phosphate dehydrogenase
G-CSF: Granulocyte colony-stimulating factor
GLUTs: Facilitated diffusion glucose transporters
GM-CSF: Granulocyte macrophage colony-stimulating factor
GPR81: G-protein-coupled receptor 81
HIF1: Hypoxia-inducible factor 1
HK: Hexokinase
HKDC1: Hexokinase domain-containing protein 1
HKs: Hexokinases
hnRNPA1: Heterogeneous nuclear ribonucleoprotein A1
hnRNPA2: Heterogeneous nuclear ribonucleoprotein A2
HRE: Hypoxia response element
IGF-1: Insulin-like growth factors -1
LDH: Lactate dehydrogenase
LDH: Lactate dehydrogenase
MCT1: Monocarboxylate transporter 1
MDSCs: Myeloid-derived suppressor cells
MMPs: Matrix metalloproteinases
MSCs: Mesenchymal stem cells
mTORC1: Mechanistic target of rapamycin complex 1
m6A: N6-methyladenosine
MSI-H: Microsatellite instability-high
MSS: Microsatellite-stable
ORR: Objective response rate
OXPHOS: Oxidative phosphorylation
PDH: Pyruvate dehydrogenase
PDK1: Pyruvate dehydrogenase kinase 1
PEP: Phosphoenolpyruvate
PET: Positron emission tomography
PFK-1: Phosphofructokinase1
PFKFB/PFK2: 6-Phospho-fructo-2-kinase/fructose-2,6-bisphosphatase
PFS: Progression-free survival
PI3K: Phosphoinositide 3 kinase
PK: Pyruvate kinase
PKL: Liver PK
PKR: Red blood cell PK
ROS: Reactive oxygen species
SGLTs: Sodium-glucose linked transporters
SRSF3: Serine/arginine-rich splicing factor 3
TAMs: Tumor-associated macrophages
TCA: Tricarboxylic acid
TECs: Tumor endothelial cells
TGIF2: TGF-β-induced factor homeobox 2
TXNIP: Thioredoxin-interacting protein
TIME: Tumor immune microenvironment
TIMs: Tumor-infiltrating myeloid cells
TME: Tumor microenvironment
TOP1: Topoisomerase 1
Treg: Regulatory T cell
VEGF: Vascular endothelial growth factor
VHL: Von Hippel–Lindau protein
